# Selected Personality Traits and Employment as the Predictors of the Intensity of Psychosocial Problems Experienced by Chronically Ill Patients

**DOI:** 10.3390/ijerph20010747

**Published:** 2022-12-31

**Authors:** Wojciech Otrębski, Katarzyna Mariańczyk, Karolina Krzysztofik

**Affiliations:** Institute of Psychology, John Paul II Catholic University of Lublin, 20-950 Lublin, Poland

**Keywords:** chronic illness, psychosocial problems, occupational activity, personal resources

## Abstract

Background. This study sought to determine the effect of being employed or unemployed on the relationships between selected personal variables (acceptance of one’s illness, self-efficacy, and self-actualization) and the intensity of psychosocial problems experienced by chronically ill persons (ChIP). Participants and procedures. The PCH-R Scale, the Generalized Self-Efficacy Scale, the Acceptance-of-Illness Scale, and the Self-Actualization Scale were used to collect information from 236 employed and unemployed ChIP. Results. Acceptance of illness and self-efficacy predicted the intensity of general psychosocial problems and problems in the personal, family, social, and occupational spheres in both employed and unemployed ChIP. Conclusions. The results can significantly contribute to increasing the effectiveness of treatment and support offered to ChIP. Continued employment or reemployment after a period of intense therapy can protect them from a rapid degradation of their personal—and frequently, socio-economic—resources, which are necessary for them to be able to adapt to new challenges and maintain a good quality of life, despite experiencing a chronic illness.

## 1. Introduction

Chronic illness was not seen as a major socio-economic and health issue until the 20th century. According to The National Center for Chronic Disease Prevention and Health Promotion (NCCDPHP), a chronic illness is one that “lasts 1 year or more and requires ongoing medical attention or limit activities of daily living or both” [1]. As chronic illnesses have different natural histories and clinical and social consequences (the latter depending on whether or not the symptoms are visible), the psychosocial problems experienced by people affected by them are different too. Chronic illnesses frequently cause people to change their lifestyles, struggle with discomfort and pain, abandon or adjust their social roles, strive to maintain financial stability, seek different employment, or rethink their careers. People affected by chronic illnesses are also more dependent on family and community support [2,3,4,5,6].

The magnitude of the socio-economic impact of chronic illnesses is well illustrated by recent Eurostat data, according to which the proportion of people diagnosed with long-term illnesses increased in Europe from 24% in 2010 to 30% in 2017 [7]. Chronic illnesses place special requirements, not only on individual patients, their workplace functioning, and their families, but also on their national economies [8,9,10,11,12].

The share of people with chronic illnesses among Poland’s 5.5 million economically inactive population is also rising, as Statistics Poland’s estimates show [13]. A 2017 survey found that 41% of Poles feared developing a chronic illness and becoming a burden for their families, 30% were concerned about having an illness that would make them unable to work and pay for their basic needs, and 25% worried about not having money to afford effective treatment [14]. 

Developing a chronic illness is a life-altering experience, as the awareness of having it and its prolonged or permanent implications redefine the physical, mental, family, social, and occupational aspects of the affected person’s life [6,15,16]. Some authors call this change revolutionary [17,18], emphasizing that a chronic illness impairs quality of life, brings on many challenges, increases the risk of chronic stress, and necessitates adaptation to new and adverse circumstances [19,20,21].

The psychophysical and social well-being of individuals facing life challenges is influenced by various personal, social, cultural, and other resources [22,23,24]. Among these is employment, which is assigned a special role in preventing the chronic illness from causing the inability to work [10,25,26]. In Kowalik’s opinion [27], staying employed, seeking a new job, and withdrawing from the labor market are ways of coping with a chronic illness. For people faced with long-term treatment due to chronic illness, employment is as important as it is for people with disabilities: it is part of their daily struggle for normalcy and preserving the pre-illness rhythm of life. In many cases, it is even a reason why they want to live and undergo treatment and is an essential element of their economic safety [27,28,29,30].

The ability to cope with a chronic illness is mainly attributed to the possession of personal resources, such as a sense of coherence, a healthy locus of control, optimism, a sense of self-efficacy, a sense of self-esteem, hope, a sense of humor, emotional intelligence, and personality [22,24,27,31,32,33,34]. 

Therefore, the identification and development of personal resources can be a way to enhance a person’s health potential. According to research, the belief in self-efficacy significantly contributes to patients accepting treatment and effectively coping with chronic conditions such as chronic obstructive pulmonary disease [31,35], diabetes, cardiac diseases, and asthma [36].

There is also evidence pointing to a relationship between patients’ acceptance of illness and the quality of their lives: greater acceptance of illness improves patients’ adaptation to it and lowers discomfort [31].

## 2. Method

**Figure 1:**
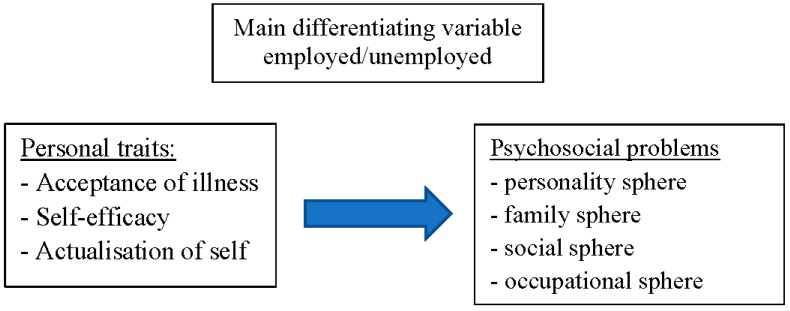
The theoretical design of the study.

The main purpose of this study was to determine how being employed or unemployed influences relationships between selected personal variables (acceptance of illness, self-efficacy, and self-actualization) and the intensity of psychosocial problems experienced by chronically ill persons (Figure 1).

Based on the literature pertinent to the subject of the study, the following hypotheses were formulated:

**Hypothesis 1.**  *Acceptance of illness, self-efficacy, and self-actualization are greater in employed chronically ill persons than in those who are unemployed.*

**Hypothesis 2.**  *Employed chronically ill persons experience a lower intensity of psychosocial problems compared with those who do not have jobs.*

**Hypothesis 3.**  *Acceptance of illness, self-efficacy, and self-actualization of chronically ill persons are significantly and negatively related to the intensity of psychosocial problems they experience.*

**Hypothesis 4.**  *The personality traits of chronically ill persons (i.e., acceptance of illness, self-efficacy, and self-actualization) determine the intensity of their psychosocial problems.*

### 2.1. Participants 

Participants were selected for the study on a voluntary basis using purposive sampling. Their contact data were obtained from social media, internet forums, medical centers, and clinics. Eligibility for the study was assessed based on whether a person was chronically ill and had or did not have a job. All participants were assured that the study had a scientific purpose and that their participation would be anonymized.

A total of 236 chronically ill persons were interviewed for the study. Depending on their labor market status, they were assigned to the E (employed) subgroup or the UE (unemployed) subgroup. The E subgroup consisted of 136 persons at a mean age (M) of 44.63 years, of whom 61.00% were female and 39.00% were male. The UE subgroup included 100 participants at a mean age of 43.83 years, with females and males accounting for 56.00 and 44.00%, respectively.

Regarding education levels, 69% of the participants in the E subgroup and 34% in the UE subgroup had received higher education. Most participants (66.50% in the E and 56.00% in the UE) lived in big and medium-sized cities, around 10% came from small towns, and more than 20% were rural residents (23.50% in the E subgroup and 32.00% in the UE subgroups).

Cardiovascular patients accounted for 27.30% of the sample, 21.61% of the participants had diabetes, 13.98% were diagnosed with thyroid disease, and 9.32% had neoplasm; the other 27.79% suffered from various diseases, such as multiple sclerosis, asthma, irritable bowel syndrome, and spinal disorders; the average illness duration in the group was M = 10.47 years. Most participants (46.61%) learned about their chronic condition 6 years prior to the study, 22.04% 6 to 10 years prior, 19.91% 10 to 20 years prior, and 11.44% had endured their illness for longer than 20 years. 

In the E subgroup, 80.15% of the participants were helped by others in coping with their illness, compared with 69.00% in the UE subgroup. Almost all participants (91.18%) who had jobs were satisfied with them; of those, 88% wished to continue employment. More than one-third (40.44%) believed that employment benefitted their health, 32.36% were unsure of whether it had any effect on it, and 27.20% thought that employment was detrimental to their health. In the UE subgroup, 63% of the participants wanted to work, but only 39.00% made an effort to find a job; almost all of them (85.00%) had worked previously. Chronic illness was indicated as the cause of joblessness in 42% of the unemployed participants; the other 58% did not think it influenced their situation. Assessing the effect of unemployment on their health, half of the jobless participants stated that it had no effect, 30% thought that not having a job benefitted their health, and 20% considered that it was detrimental to it.

### 2.2. Research Tools

The study was conducted using five research tools: the PCH-R Scale [37], the Generalized Self-Efficacy Scale (GSES) [38], the Acceptance-of-Illness Scale (AIS) [38,39], the Actualization-of-Self Scale (AS-5-R) [37], and the Demographic Data Inventory.

The PCH-R was designed by Witkowski, Mariańczyk, Otrębski, and Wiącek [37] to assess the intensity of chronically ill persons’ psychosocial problems in the personality (PP-P), family (PP-F), social (PP-S), and occupational (PP-O) spheres. Each sphere has 60 items rated on scales from 0–5. The general PCH-R score (PP-G) and the scores for the individual spheres are calculated. A higher general score is interpreted as indicating a greater intensity of psychosocial problems and a stronger sense of disability. The PCH-R has a Cronbach’s alpha of 0.95 and uses sten norms. 

The GSES developed by Schwarzer and Jerusalem [38] is applied to determine a person’s belief in their self-efficacy understood as an ability to deal with challenges and problems. Its Polish version was prepared by Juczyński in 2001. The GSES has 10 items rated on a 4-point scale, so the minimum score is 10 and the maximum score is 40; a high score indicates a stronger belief in one’s self-efficacy. The Polish GSES Cronbach’s alpha is 0.85. 

Felton et al.’s AIS [39] measures adult patients’ acceptance of illness and the degree to which they have learned to live with it. Its Polish version was prepared by Juczyński [38]. The AIS has 8 items measured on scales from 1–5, where 1 means “I strongly agree,” and 5 means “I strongly disagree.” Therefore, the minimum score achievable is 8, and the maximum is 40. A higher score points to a greater acceptance of illness. As in its original version, the AIS has a Cronbach’s alpha of 0.85 and a test-retest reliability of 0.64.

The AS-5-R, created by Witkowski, Wiącek, and Otrębski [37], helps to determine a person’s level of self-actualization, understood as a purposeful and conscious process of self-development. The AS-5-R has 16 bipolar scales for measuring 16 aspects of the process: setting life goals, realistic attitude, avoidance of dichotomous thinking, time perspective, belief in general human values, non-labeling of others, capacity for emotional contact with others, self-acceptance, focusing on tasks rather than on defending oneself, internal locus of control, occasional need for seclusion, depth of emotional experiences, thinking for oneself instead of conforming to others’ opinions, a sense of humor, and creativity. Each aspect is assessed on a scale from 1–7, where 1 means self-actualization, and 7 indicates its absence. A person’s level of self-actualization is determined based on the general score (AS-G) and the scores for the following spheres: attitude toward reality (AS-R), attitude toward others (AS-P), self-perception (AS-Y), and self-expression (AS-E). The AS-5-R has a Cronbach’s alpha of 0.72 and uses sten norms for the general score.

### 2.3. Procedure

Research data were gathered in the course of face to face interviews. Before they commenced, participants were advised on the purpose of the interview, and that they were free to withdraw from it at their discretion. The study was designed in conformity with the Declaration of Helsinki.

### 2.4. Data Analysis

Statistical analysis was performed using SPSS software. The investigated variables were described using means and standard deviations, as well as frequency and percentage distributions. The between-group differences in variable values were assessed using *t*-tests and the measurement of effect size (Cohen’s d). The between-variable correlations were assessed by Pearson’s *r* coefficient. Hypothesis 4 was tested in the theoretical linear regression framework. A variable’s weight in the equation was determined using *β* (a standardized regression coefficient) and *R*^2^ (a model fit coefficient).

## 3. Results

### 3.1. The Results of Statistical Analysis

The analysis of the personal variables showed average acceptance of illness (M = 30.55; M = 28.12) and self-efficacy (M = 29.89; M = 29.66), and a high level of self-actualization (M = 89.86; M = 84.56) in the group. Statistically significant differences between employed and unemployed participants, with an average effect size, were only found for acceptance of illness (*p* ≤ 0.05) and general self-actualization (*p* ≤ 0.01); both were higher among the employed participants (Table 1).

The intensity of participants’ psychosocial problems was low both regarding general problems (M = 101.45; M = 118.25) and problems in each of the four spheres: PP-P (M = 29.69; M = 32.67); PP-F (M = 23.96; M = 28.60); PP-S (M = 24.28; M = 28.39); and PP-O (M = 23.57; M = 28.59). Statistically significant differences between the subgroups in the intensity of psychosocial problems and an average effect size were determined for the general score (*p* ≤ 0.05) and the scores for the family and occupational spheres (*p* ≤ 0.05). This implies that the intensity of general psychosocial problems and problems in the family and occupational spheres is lower in the case of chronically ill persons with jobs than in those who are not employed (Table 1).

### 3.2. Correlations between the Variables

In both subgroups, the explanatory variables (acceptance of illness, self-efficacy, and self-actualization) were inversely related to the explained variable (psychosocial problems). As many as 27 of 35 correlation coefficients in the E subgroup and less than half (16 of 35) of those obtained for the UE group were statistically significant (Table 2 and Table 3).

The unemployed participants’ acceptance of illness and self-efficacy were significantly, negatively, and moderately correlated with the intensity of general psychosocial problems (*p* ≤ 0.01) and problems in their four spheres (PP-P *p* ≤ 0.01; PP-F *p* ≤ 0.01; PP-S *p* ≤ 0.01; PP-O *p* ≤ 0.01). Self-actualization was significantly, positively, and weakly correlated with the intensity of general psychosocial problems (*p* ≤ 0.05) and problems in three spheres (PP-F *p* ≤ 0.05; PP-S *p* ≤ 0.05; PP-O *p* ≤ 0.01). The reality sphere and the self-perception sphere of self-actualization proved to be significantly, negatively, and weakly correlated with the intensity of general psychosocial problems (*p* ≤ 0.05) and problems in their four spheres (PP-P *p* ≤ 0.05; PP-F *p* ≤ 0.05; PP-S *p* ≤ 0.05; PP-O *p* ≤ 0.05). Significant, negative, and weak correlations were also determined between the self-expression sphere and the intensity of general psychosocial problems (*p* ≤ 0.05) and two of their spheres (PP-S *p* ≤ 0.05; PP-O *p* ≤ 0.01). This indicates that in employed chronically ill persons, greater acceptance of illness, self-efficacy, and self-actualization are associated with a lower intensity of psychosocial problems (Table 2).

The unemployed participants’ levels of acceptance of illness and self-efficacy were significantly, negatively, and moderately correlated with the intensity of general psychosocial problems (*p* ≤ 0.01) and problems in their four spheres (PP-P *p* ≤ 0.01; PP-F *p* ≤ 0.01; PP-S *p* ≤ 0.01; PP-O *p* ≤ 0.01). Self-actualization was only correlated, significantly, positively and weakly, with the intensity of psychosocial problems in the occupational sphere (*p* ≤ 0.05). Regarding the self-actualization spheres, the reality sphere was found to be significantly, negatively, and weakly correlated with the intensity of general psychosocial problems (*p* ≤ 0.05) and problems in their three spheres (PP-P *p* ≤ 0.05; PP-S *p* ≤ 0.05; PP-O *p* ≤ 0.05). The self-expression sphere was significantly, negatively, and weakly correlated only with the social sphere of psychosocial problems (*p* ≤ 0.05). Therefore, greater acceptance of illness, self-efficacy, and self-actualization (in the selected spheres) appear to be associated with unemployed, chronically ill persons perceiving psychosocial problems as less intense (Table 3).

### 3.3. Predictors of the Intensity of Psychosocial Problems Experienced by Employed and Unemployed Chronically Ill Persons

The intensity of general psychosocial problems and problems in their four spheres, experienced by the E subgroup, proved to be related to acceptance of illness and sense of self-efficacy. The stronger they were, the lower the intensity of psychosocial problems observed. Regarding the level of self-actualization, no relationship was determined between its level and the intensity of psychosocial problems (Table 4).

An association between acceptance of illness and sense of self-efficacy and the intensity of general psychosocial problems and problems in their four spheres was also found in the UE subgroup. Greater acceptance of illness and a sense of self-efficacy were associated with a lower intensity of psychosocial problems; but again, no relationship was found between the level of self-actualization and the intensity of psychosocial problems (Table 5).

## 4. Discussion

The results of our research partially support all four hypotheses. Hypotheses 1, assuming that employed chronically ill persons have greater acceptance of illness, self-efficacy, and self-actualization than those without jobs was only supported with respect to acceptance of illness and self-actualization, which were statistically significantly stronger in the E subgroup.

Hypothesis 2, predicting a lower intensity of psychosocial problems in employed chronically ill persons, was only confirmed for general psychosocial problems and psychological problems in the family and occupational spheres (two of the three analyzed), which were statistically significantly more intense in the unemployed participants.

The above findings underscore the importance of having a job as a factor enabling people with chronic health conditions to adjust to the implications of their circumstances. Although negative experiences and psychosocial problems are an inevitable element of chronic illnesses and treatment, maintaining employment, or resuming work as soon as possible, can significantly reduce their intensity [3,6,18,20,36,40].

Hypothesis 3, assuming a significant and negative relationship between the intensity of psychosocial problems experienced by employed and unemployed chronically ill persons and their personality traits, such as acceptance of illness, self-efficacy, and self-actualization, and Hypothesis 4 suggesting the traits’ influence on the intensity of psychosocial problems were also confirmed only in part. All correlations between the explanatory variables and the explained variable proved negative. A markedly higher number of low or moderate statistically significant correlations in the employed subgroup than in the unemployed subgroup implied that stronger personality traits, especially acceptance of illness and self-efficacy, but also self-actualization, protected chronically ill persons from experiencing intense psychosocial problems.

The intensity of general psychosocial problems and problems in the personal, family, social, and occupational spheres was predicted by the acceptance of illness and self-efficacy in both employed and unemployed participants. Therefore, having or not having a job did not determine the set of personality traits predicting the intensity of psychosocial problems felt by chronically ill persons. Somewhat surprisingly, slightly higher *R*^2^ values obtained for the unemployed participants suggested that acceptance of illness and self-efficacy were marginally stronger predictors of the intensity of psychosocial problems in chronically ill participants who did not have jobs.

The testing results regarding the four hypotheses can significantly contribute to increasing the effectiveness of treatment and support offered to people affected by chronic health conditions. Enabling them to continue employment or resume work soon after completing intense therapy can prevent the rapid depletion of their intrinsic and extrinsic resources, facilitate their ability to adapt to new challenges, and ensure a good quality of life, despite their illness [23,28,29,33,41,42].

## Figures and Tables

**Table 1:** Values of the investigated variables by subgroup.

	Employed Participants M (*SD*)	Unemployed Participants M (*SD*)	*Df*	*t*	*d*
*AIS*	30.55 (8.28)	28.12 (8.27)	233	2.33 *	0.29
*GSES*	29.89 (4.41)	29.66 (5.25)	234	0.35	-
*AS-G*	89.86 (16.59)	84.56 (16.13)	233	2.44 **	0.32
*AS-R*	19.33 (3.47)	18.55 (4.07)	234	1.59	-
*AS-P*	20.14 (4.17)	19.98 (4.50)	234	0.29	-
*AS-Y*	20.40 (4.34)	19.23 (5.01)	234	1.92	-
*AS-E*	16.72 (3.99)	16.23 (4.17)	234	0.98	-
*PP-G*	101.49 (64.67)	118.25 (67.06)	234	−1.93 *	−0.25
*PP-P*	29.69 (17.49)	32.67 (18.02)	234	−1.27	-
*PP-F*	23.96 (17.03)	28.60 (18.13)	234	−2.01 *	−0.26
*PP-S*	24.28 (16.86)	28.39 (17.50)	234	−1.82	-
*PP-O*	23.57 (16.44)	28.59 (17.64)	234	−2.25 *	−0.30

*t*—test statistic; *AIS*—acceptance of illness; *GSES*—self-efficacy; *AS-G*—general score for self-actualization; *AS-R*—self-actualization in the reality sphere; *AS-P*—self-actualization in the social sphere; *AS-Y*—self-actualization in self-perception; *AS-E*—self-actualization in self-expression; *PP-G*—general score for psychosocial problems; *PP-P*—psychosocial problems in the personality sphere; *PP-F*—psychosocial problems in the family sphere; *PP-S*—psychosocial problems in the social sphere; *PP-O*—psychosocial problems in the occupational sphere; *df*—the number of degrees of freedom; *d*—Cohen’s *d*; ** *p* ≤ 0.01, * *p* ≤ 0.05.

**Table 2:** Between-variable correlations—the E subgroup.

	*PP-P*	*PP-F*	*PP-S*	*PP-O*	*PP-G*
*AIS*	−0.37 **	−0.42 **	−0.36 **	−0.44 **	−0.41 **
*GSES*	−0.34 **	−0.29 **	−0.27 **	−0.30 **	−0.32 **
*AS-G*	−0.12	−0.20 *	−0.17 *	−0.22 **	−0.19 *
*AS-R*	−0.21 *	−0.22 *	−0.21 *	−0.18 *	−0.21 *
*AS-P*	−0.09	−0.12	−0.13	−0.16	−0.13
*AS-Y*	−0.18 *	−0.21 *	−0.18 *	−0.20 *	−0.20 *
*AS-E*	−0.15	−0.20	−0.18 *	−0.25 **	−0.20 *

*AIS*—acceptance of illness; *GSES*—self-efficacy; *AS-G*—general score for self-actualization; *AS-R*—self-actualization in the reality sphere; *AS-P*—self-actualization in the social sphere; *AS-Y*—self-actualization in self-perception; *AS-E*—self-actualization in self-expression; *PP-G*—general score for psychosocial problems; *PP-P*—psychosocial problems in the personality sphere; *PP-F*—psychosocial problems in the family sphere; *PP-S*—psychosocial problems in the social sphere; *PP-O*—psychosocial problems in the occupational sphere; ** *p* ≤ 0.01; * *p* ≤ 0.05.

**Table 3:** Between-variable correlations—the UE subgroup.

	*PP-P*	*PP-F*	*PP-S*	*PP-O*	*PP-G*
*AIS*	−0.48 **	−0.58 **	−0.46 **	−0.45 **	−0.53 **
*GSES*	−0.35 **	−0.43 **	−0.37 **	−0.35 **	−0.40 **
*AS-G*	−0.08	−0.11	−0.19	−0.20 *	−0.15
*AS-R*	−0.20 *	−0.19	−0.28 **	−0.22 **	−0.24 *
*AS-P*	−0.04	−0.10	−0.16	−0.14	−0.12
*AS-Y*	−0.08	−0.11	−0.21 *	−0.15	−0.15
*AS-E*	−0.05	−0.09	−0.11	−0.16	−0.11

*AIS*—acceptance of illness; *GSES*—self-efficacy; *AS-G*—general score for self-actualization; *AS-R*—self-actualization in the reality sphere; *AS-P*—self-actualization in the social sphere; *AS-Y*—self-actualization in self-perception; *AS-E*—self-actualization in self-expression; *PP-G*—general score for psychosocial problems; *PP-P*—psychosocial problems in the personality sphere; *PP-F*—psychosocial problems in the family sphere; *PP-S*—psychosocial problems in the social sphere; *PP-O*—psychosocial problems in the occupational sphere; ** *p* ≤ 0.01; * *p* ≤ 0.05.

**Table 4:** Predictors of psychosocial problems—the E subgroup.

Explained Variable: *PP-G*				
*R* ^2^		*F*		*P*
0.21		7.33		0.008
	*β*	*t*	*p*	95% CI
Constant	279.96 (B)	7.89	<0.001	209.84:350.09
AIS	−0.35	−4.27	<0.001	−3.95:−1.45
GSES	−0.22	−2.70	0.008	−5.59:−0.87
Explained variable: *PP-P*				
*R* ^2^		*F*		*P*
0.19		9.54		0.002
	*β*	*t*	*p*	95% CI
Constant	78.54 (B)	8.06	<0.001	59.28:97.79
AIS	−0.29	−3.52	<0.001	−0.95:−0.26
GSES	−0.25	−3.09	0.002	−1.66:−0.36
Explained variable: *PP-F*				
*R* ^2^		*F*		*P*
0.21		5.86		0.017
	*β*	*t*	*p*	95% CI
Constant	69.10 (B)	7.36	<0.001	50.54:87.65
AIS	−0.36	−4.39	<0.001	−1.06:−0.40
GSES	−0.20	−2.42	0.017	−1.39:−0.14
Explained variable: *PP-S*				
*R* ^2^		*F*		*P*
0.16		5.31		0.023
	*β*	*t*	*p*	95% CI
Constant	64.89 (B)	6.80	<0.001	46.02:83.76
AIS	−0.30	−3.59	<0.001	−0.94:−0.27
GSES	−0.19	−2.30	0.023	−1.37:−0.10
Explained variable: *PP-O*				
*R* ^2^		*F*		*P*
0.22		5.61		0.19
	*β*	*t*	*p*	95% CI
Constant	67.43 (B)	7.52	<0.001	49.70:85.15
AIS	−0.37	−4.65	<0.001	−1.06:−0.42
GSES	−0.19	−2.39	0.019	−1.31:−0.11

*R*^2^—model fit coefficient; *t*—test statistic; *β*—standardized regression coefficient; *p*—statistical significance; *AIS*—acceptance of illness; *GSES*—self-efficacy; *PP-G*—general score for psychosocial problems; *PP-P*—psychosocial problems in the personality sphere; *PP-F*—psychosocial problems in the family sphere; *PP-S*—psychosocial problems in the social sphere; *PP-O*—psychosocial problems in the occupational sphere.

**Table 5:** Predictors of psychosocial problems—the UE subgroup.

Explained Variable: *PP-G*				
*R* ^2^		*F*		*P*
0.31		8.38		0.005
	*β*	*t*	*p*	95% CI
Constant	314.02 (B)	9.43	<0.001	247.93:380.11
AIS	−0.44	−4.98	<0.001	−4.96:−2.13
GSES	−0.25	−2.89	0.005	−5.47:−1.02
Explained variable: *PP-P*				
*R* ^2^		*F*		*P*
0.27		5.44		0.022
	*β*	*t*	*p*	95% CI
Constant	79.29 (B)	8.50	<0.001	60.78:97.81
AIS	−0.41	−4.45	<0.001	−1.28:−0.49
GSES	−0.21	−2.23	0.022	−1.35:−0.10
Explained variable: *PP-F*				
*R* ^2^		*F*		*P*
0.39		10.73		0.001
	*β*	*t*	*p*	95% CI
Constant	86.41 (B)	10.10	<0.001	69.44:103.39
AIS	−0.48	−5.80	<0.001	−1.42:−0.70
GSES	−0.27	−3.27	0.001	−1.51:−0.37
Explained variable: *PP-S*				
*R* ^2^		*F*		*P*
0.26		6.95		0.010
	*β*	*t*	*p*	95% CI
Constant	74.92 (B)	8.20	<0.001	56.79:93.05
AIS	−0.37	−4.09	<0.001	−1.19:−0.41
GSES	−0.24	−2.63	0.010	−1.42:−0.20
Explained variable: *PP-O*				
*R* ^2^		*F*		*P*
0.25		6.04		0.016
	*β*	*t*	*p*	95% CI
Constant	73.38 (B)	8.00	<0.001	55.19:91.57
AIS	−0.37	−4.06	<0.001	−1.18:−0.40
GSES	−0.22	−2.45	0.016	−1.37:−0.14

*R*^2^—model fit coefficient; *t*—test statistic; *β*—standardized regression coefficient; *p*—statistical significance; *AIS*—acceptance of illness; *GSES*—self-efficacy; *PP-G*—general score for psychosocial problems; *PP-P*—psychosocial problems in the personality sphere; *PP-F*—psychosocial problems in the family sphere; *PP-S*—psychosocial problems in the social sphere; *PP-O*—psychosocial problems in the occupational sphere.

## Data Availability

Data confirming the obtained and presented results are available from the authors of the article. If necessary, please contact the authors by email.
